# Patient, Provider, and Health System Determinants of Hospice Length of Stay

**DOI:** 10.1089/pmr.2024.0077

**Published:** 2025-04-03

**Authors:** Eliza Thompson, Daniel Sanchez Pellecer, Gregory J. Hanson, Shealeigh A. Inselman, Jenn M. Manggaard, Kevin J. Whitford, Jacob J. Strand, Rozalina G. McCoy

**Affiliations:** ^1^ Department of Medicine, Mayo Clinic School of Graduate Medical Education, Rochester, Minnesota, USA.; ^2^ Division of Community Internal Medicine, Geriatrics, and Palliative Care, Department of Medicine, Mayo Clinic, Rochester, Minnesota, USA.; ^3^ Robert D. and Patricia E. Kern Center for the Science of Health Care Delivery, Mayo Clinic, Rochester, Minnesota, USA.; ^4^ Division of Hospital Internal Medicine, Department of Medicine, Mayo Clinic, Rochester, Minnesota, USA.; ^5^ Division of Endocrinology, Diabetes, and Nutrition, Department of Medicine, University of Maryland School of Medicine, Baltimore, Maryland, USA.; ^6^ Department of Medicine, Division of Endocrinology, Diabetes, and Nutrition, Institute for Health Computing, University of Maryland, Bethesda, Maryland, USA.

**Keywords:** hospice, length of stay, palliative, referral

## Abstract

**Background:**

Benefits of hospice care, such as improvement in quality of life and reduced costs, depend on duration of enrollment in hospice services, making timely hospice referral essential. Patient-, provider-, and system-level factors associated with hospice referral timing and length of stay (LOS) are unclear.^1–6^

**Objective:**

To review existing hospice referral patterns to identify patient- and system-level factors associated with hospice LOS.

**Methods:**

We performed a retrospective review of all adult patients referred from our institution (located in Rochester, Minnesota, USA) to a nonprofit hospice agency between 2013 and 2017. The primary dependent variable was hospice LOS. Patient-level variables included demographic characteristics, place of residence, and hospice diagnosis. System-level variables included referral setting and provider-specific variables, such as title and gender. Statistical analyses were performed using multivariate logistic regression.

**Results:**

A total of 2072 patients were referred to hospice during the study period. Patient-level factors associated with LOS included hospice diagnosis and place of residence. Patients referred for cancer had a higher odds of a long LOS, while patients from long-term care facilities had a higher odds of a short LOS. System-level factors associated with LOS included provider gender and title. Referral by a female provider or by a physician, rather than an advanced practice provider, was associated with a lower odds of a short LOS.

**Conclusion:**

Based on a review of hospice referral patterns, the integration of hospice care into subspecialty practices, long-term care facilities, and advanced practice education could be an effective strategy to improve hospice LOS.

## Key Message

A review of 2072 hospice referrals demonstrates that patients who are residents of long-term care facilities, have a nurse practitioner or physician assistant as referring provider, are referred by a male provider, or have a diagnosis other than cancer have greater odds of experiencing a short hospice length of stay.

## Introduction

The U.S. and world populations are aging rapidly, increasing the prevalence of multimorbidity, frailty, and functional dependence with great impact on patients, caregivers, communities, health systems, and society. As a patient’s goals and preferences for their care change, clinicians must adapt therapeutic plans and care delivery models to ensure appropriate symptom and disease management throughout the trajectory of the patient’s illness.^1^ At the end of life, hospice offers a patient-centered approach to care that prioritizes symptom management, patient comfort, and goal-concordant care. Hospice improves the quality of life for patients and their caregivers while simultaneously decreasing health care costs.^2^ Importantly, the benefits of hospice are directly related to the duration of enrollment in hospice services.^3^ Time is needed to establish an individualized care plan grounded in the understanding of the physical, psychological, and spiritual needs of patients and families, as well as to reap the cost savings of foregoing potentially futile interventions and treatments. Thus, shorter hospice length of stay (LOS) has been linked to worse caregiver satisfaction, fewer patient services delivered at the end of life, and greater probability of dying in the hospital rather than at home.^4–6^ Despite the acknowledged benefits of timely hospice enrollment—and longer hospice LOS—current estimates reveal that nearly 27.9% of hospice enrollees in the United States stay in the program for fewer than seven days.^7^

Improving the timeliness of hospice enrollment and thereby lengthening hospice LOS has been identified as a priority by the National Quality Forum.^8^ The American Society of Clinical Oncology (ASCO) similarly recommends integrating palliative care services early during any given cancer diagnosis.^9^ As a result of these efforts, as well as continued focus on quality of life and goal-concordant care, the National Hospice and Palliative Care Organization (NHPCO) reported an increase in hospice utilization by more than 20% by 2018 (1.48 million new admissions) in comparison with 2014 (1.19 million new admissions). However, even though hospice enrollment has increased, LOS appears to be decreasing over time. The NHPCO reported that while longer LOS in hospice are desired, the median hospice LOS in the U.S. has fallen from 29 days in 1995 to 26 days in 2005 and just 18 days in 2018.^10^

Identifying factors associated with short hospice LOS and understanding the potential barriers to timely referral are therefore critical to improving the quality of care for patients at the end of life. Quantitative data regarding hospice referral practices are limited, as existing investigations of hospice referral processes are largely limited to surveys of patient and provider perceptions.^3,5,11^ While informative, these data are difficult to generalize. In this study, we sought to identify patient- and system-level factors associated with short length of hospice stay among all patients enrolled in nonprofit hospice centers in Olmsted County, Minnesota, USA, after referral by Mayo Clinic inpatient and outpatient providers between 2013 and 2017.

## Methods

### Study design and setting

After obtaining approval from the Mayo Clinic Institutional Review Board, we performed a retrospective analysis of all adult patients referred to hospice by a Mayo Clinic provider in Rochester, Minnesota, and enrolled in one of two area nonprofit hospice agencies between January 1, 2013, and December 31, 2017.

Mayo Clinic is an integrated health care delivery system delivering primary and specialty care to local, national, and international patients with a central hub in Rochester, Minnesota. Mayo Clinic, Rochester, hospitals have over 2000 licensed beds. The Division of Community Internal Medicine, Geriatrics, and Palliative Care (CIMGP) oversees the care for Mayo Clinic employees and their dependents, local area community-dwelling residents, residents of area assisted living and skilled nursing facilities, as well as patients receiving palliative care. In contrast to the community and geriatrics practices, which provide longitudinal primary care to area patients, the Palliative Care section delivers consultative care to local as well as national/international patients in both the ambulatory and inpatient settings. The Geriatrics section within CIMGP administers a Palliative Care Homebound Program (PCHP), an innovative health delivery strategy used to provide patient-centered care for patients with high illness burden and functional decline.^12^ This program is designed as a bridge service providing home-based primary care in the months or years preceding hospice eligibility.

We focused specifically on referrals made to two large area hospice agencies, Mayo Hospice and Seasons Hospice. These hospice agencies are the only nonprofit agencies in the area and make up the vast majority of referrals made from Mayo Clinic. Mayo Hospice, a Medicare-certified hospice program since 1980, is affiliated with Mayo Clinic but is overseen by an independent governing board and medical directors. Seasons Hospice is a community-based hospice program with both residential and outpatient services operating in the Rochester, Minnesota, community since 1996.

### Study population

We included all adult (age ≥18 years) patients who were referred to either Mayo Hospice or Seasons Hospice by a Mayo Clinic provider between January 1, 2013, and December 31, 2017. Patients were identified from the records kept by the two hospice agencies. Patients who were discharged from hospice alive or transferred to a different hospice agency were excluded from analysis. Patients who transferred between the two included hospice agencies were included if enrollment was continuous, with LOS determined by the sum of stay in the two agencies. The most common transfer was from Mayo Hospice to Seasons Hospice, driven by Seasons Hospice’s access to an inpatient hospice house; Mayo Hospice does not have an inpatient facility.

### Independent and dependent variables

For each patient, Mayo Hospice and Seasons Hospice provided basic information on the dates of admission and discharge; whether the patient was discharged alive, transferred, or died; and the hospice admitting diagnosis. Mayo Clinic electronic health records of each patient were reviewed to extract demographic, clinical, and provider characteristics detailed below.

The primary dependent variable was hospice LOS, calculated using the dates of admission and discharge provided by hospice institutions. The LOS was assessed as a continuous variable and stratified into categories, short and long, to allow for assessment of odds ratios. Short LOS was defined as up to 7 days, while a long LOS was defined as greater than 90 days. These thresholds were selected based on existing literature and CMS billing patterns.^7^

Both patient-level and system-level independent variables were investigated for their association with short or long LOS. Patient-level variables included demographic characteristics (age, sex, race, ethnicity, marital status, and language), hospice diagnosis, and place of residence prior to enrollment (home, long-term care facility, assisted living facility). Race, ethnicity, and sex were examined to assess for disparities in hospice referral patterns in populations with known gaps in the receipt of palliative care.^13,14^ We categorized hospice diagnoses as reported to CMS (cancer, heart disease, dementia, lung disease, stroke, or other).

System-level variables included referral source and referring provider characteristics. Referral source was defined as inpatient, outpatient, or specialty care program. The specialty care program included those patients referred from the palliative homebound program, described above. Provider-specific variables included (1) referring clinician type, classified as attending physician, trainee physician (resident or fellow), or advanced practice provider (i.e., nurse practitioner [NP] or physician assistant [PA]) and (2) gender, classified as male, female, or team. An individual referring provider could only be accurately identified for patients referred from the outpatient setting, as patients referred from the inpatient setting were referred by their treating team.

### Statistical analysis

We described our cohort’s demographic, clinical, and system-based attributes using counts (proportions) for categorical variables and means (standard deviation [SD]) or medians (interquartile range [IQR]) for continuous variables. For each hospice diagnosis, as well as the overall cohort, we calculated the median LOS in days. To identify factors associated with short and long hospice LOS (≤7 days and >90 days, respectively), we used mixed-effect logistic regression to calculate odds ratios (OR; 95% confidence intervals [CI]) for the patient-, system-, and provider-level variables, using the hospice agency as a random effect. Two-sided *p*-values <0.05 were considered statistically significant. All statistical analyses were conducted using SAS 9.04.01M6.

## Results

Between January 2013 and December 2017, 2,072 adult patients were referred to hospice and enrolled in either Mayo Hospice or Seasons Hospice (Table 1). The mean age of referred patients was 77.3 years (SD, 14.5), 50% were women, 95% were non-Hispanic White, and 55.4% were married or living with a partner. The most common hospice-qualifying diagnosis was cancer, documented in 1028 patients (49.6%). Most patients (67.4%; *N* = 1397) lived at home prior to hospice enrollment, while 16.6% (*N* = 344) resided in long-term care facilities and 16.0% (*N* = 331) in assisted living facilities (including memory care units). A similar number of referrals was initiated from the inpatient setting (44.4%; *N* = 919) as from the outpatient setting (44.3%; *N* = 918), while the remainder (11.3%; *N* = 235) were referred to hospice by the palliative care homebound interdisciplinary team.

**Table 1.** Baseline Characteristics

Baseline characteristics	Total (*N* = 2072)
Age (mean)	77.30
Patient Sex, *n* (%)	
Female	1037 (50)
Male	1037 (50)
Ethnicity/Race, *n* (%)	
Non-Hispanic White	1960 (94.6)
Non-Hispanic Black	24 (1.20)
Hispanic	17 (0.80)
Asian	33 (1.60)
Other/Unknown	38 (1.80)
Marital status, *n* (%)	
Married/Life partnership	1148 (55.40)
Other	924 (44.60)
Language, *n* (%)	
Limited English proficiency	83 (4.0)
English-speaking	1989 (96.0)
Place of residence prior to hospice enrollment, *n* (%)	
Assisted living facility (includes memory units and group homes)	331 (16.00)
Private residence	1397 (67.40)
Long-term care	344 (16.60)
Primary hospice diagnosis, *n* (%)	
Cancer	1028 (49.60)
Dementia	200 (9.70)
Heart disease	307 (14.80)
Other	275 (13.30)
Lung disease	178 (8.60)
Stroke	84 (4.10)
Referring source, *n* (%)	
Inpatient	919 (44.40)
Outpatient	918 (44.30)
Primary Care Homebound Program	235 (11.30)
Referring provider title	
Attending	614 (29.60)
NP/PA	168 (8.10)
Trainee (resident or fellow)	130 (6.30)
Team	1160 (56.00)
Referring provider gender, *n* (%)	
Male	510 (24.60)
Female	402 (19.40)
Team (inpatient)	1160 (56.00)

NP, nurse practitioner; PA, physician assistant.

Median hospice LOS was 18 days (IQR 6.0, 54.0) and varied according to hospice diagnosis (Table 2). Hospice enrollees with a primary diagnosis of heart disease had the longest median LOS (26 days, IQR 5.0, 88.0), followed by patients with lung disease (median 22.5 days, IQR 6.0, 87.0). Patients enrolled in hospice for stroke had the shortest median LOS of only 4 days (IQR, 2.0, 8.0). Overall, 650 patients (31.4%) had an LOS ≤7 days, and 316 patients (15.3%) had an LOS >90 days. Patients referred from the hospital had the shortest hospice LOS with a median of 9 days (IQR 4.0, 30.0), followed by patients referred from the outpatient setting, whose median LOS was 28 days (IQR 9.0, 67.0). Patients who transitioned to hospice while enrolled in the PCHP had the longest median hospice LOS with 43 days (IQR 14.0, 112.0).

**Table 2.** Hospice Length of Stay (in Days) by Hospice Diagnosis

Hospice diagnosis	*N* (%)	Hospice LOS (days) median (IQR)
Heart disease	307 (14.80)	26.0 (5.0, 88.0)
Lung disease	178 (8.60)	22.5 (6.0, 87.0)
Dementia	200 (9.60)	20.5 (7.0, 91.5)
Cancer	1028 (49.60)	19.5 (7.0, 45.0)
Stroke	84 (4.0)	4.0 (2.0, 8.0)
Other	275 (13.20)	14.0 (4.0, 51.0)

Tables 3 and 4 present results of multivariable analyses examining the association between key patient- and system-level factors with either short (<7 days) or long (>90 days) hospice LOS. While Asian patients had a higher odds of experiencing a long hospice LOS, there were no other associations between patient race/ethnicity, sex, marital status, or English proficiency status and hospice LOS, short or long. Higher age was associated with a marginally increased odds of a short hospice LOS (OR: 1.01, 95% CI: 1.00, 1.02). Hospice qualifying diagnosis and place of residence prior to hospice enrollment were both associated with likelihood of short and long hospice LOS. Compared with patients with cancer, the hospice diagnoses associated with greater odds of a short hospice LOS were heart disease (OR: 1.38, 95% CI: 1.0, 1.92), stroke (OR: 6.45, 95% CI: 3.78, 11.01), and other diagnoses (OR: 1.57, 95% CI: 1.15, 2.15). The reverse was also true for odds of long hospice LOS, with dementia, heart disease, respiratory, and other diagnoses being associated with significantly greater odds of a long hospice LOS than cancer. Compared with patients living at home, living at a long-term care facility prior to enrollment was associated with greater odds of short hospice LOS (OR: 1.42, 95% CI: 1.07–1.89) and lower odds of a long hospice LOS (OR: 0.62, 95% CI: 0.41–0.93).

**Table 3.** Patient and Provider Factors Associated with Short (≤7 Days) Hospice Length of Stay

	Odds ratio (95% CI)	*p*-Value
Patient-related variables		
Age	1.01 (1, 1.02)	0.045
Patient sex		
** **Male	Reference	
** **Female	0.87 (0.71, 1.08)	0.22
Race/Ethnicity		
** **Non-Hispanic White	Reference	
** **Non-Hispanic Black	1.15 (0.43, 3.06)	0.777
** **Hispanic	1.65 (0.55, 4.94)	0.372
** **Asian	0.74 (0.24, 2.25)	0.597
** **Other/Unknown	0.72 (0.32, 1.59)	0.412
Marital status		
** **Married/Life partnership	Reference	
** **Other/Unknown	0.8 (0.64, 1)	0.054
Interpreter		
** **Yes	0.73 (0.28, 1.9)	0.513
Hospice diagnosis		
** **Cancer	Reference	
** **Dementia	1.47 (0.98, 2.22)	0.062
** **Heart disease	1.38 (1, 1.92)	0.05
** **Lung disease	1.03 (0.7, 1.52)	0.889
** **Stroke	6.45 (3.78, 11.01)	<0.001
** **Other	1.57 (1.15, 2.15)	0.004
Place of residence		
** **Home	Reference	
** **ALF	0.88 (0.64, 1.22)	0.437
** **LTC	1.35 (1.01, 1.8)	0.04
Provider-related variables		
Referring source		
** **Outpatient	Reference	
** **Inpatient	2.56 (0.47, 14)	0.277
** **PCHP	0.81 (0.14, 4.66)	0.812
Referring provider gender		
** **Male	Reference	
** **Female	0.63 (0.42, 0.96)	0.03
** **Team	0.56 (0.1, 3.12)	0.51
Referring provider title		
** **Attending physician	Reference	
** **NP/PA	1.73 (1.04, 2.88)	0.036
** **Trainee (resident or fellow)	0.86 (0.51, 1.46)	0.582

CI, confidence interval; PCHP, Palliative Care Homebound Program.

**Table 4.** Patient and Provider Factors Associated with Long (>90 Days) Hospice Length of Stay

	Odds ratio (95% CI)	*p*-Value
Patient-related variables		
Age	1 (0.99, 1.02)	0.421
Patient sex		
** **Male	Reference	
** **Female	0.96 (0.73, 1.26)	0.751
Race/Ethnicity		
** **Non-Hispanic White	Reference	
** **Non-Hispanic Black	0.93 (0.2, 4.3)	0.929
** **Hispanic	1.02 (0.21, 5.09)	0.979
** **Asian	4.28 (1.37, 13.41)	0.013
** **Other/Unknown	0.79 (0.25, 2.47)	0.687
Marital status		
** **Married/Life partnership	Reference	
** **Other/Unknown	1.29 (0.97, 1.7)	0.077
Interpreter		
** **Yes	0.65 (0.2, 2.14)	0.48
Hospice diagnosis		
** **Cancer	Reference	
** **Dementia	2.34 (1.49, 3.7)	<0.001
** **Heart disease	2.47 (1.67, 3.66)	<0.001
** **Lung disease	3.21 (2.09, 4.93)	<0.001
** **Stroke	0.49 (0.17, 1.42)	0.187
** **Other	2.05 (1.37, 3.09)	0.001
Place of residence		
** **Home	Reference	
** **ALF	0.95 (0.67, 1.36)	0.799
** **LTC	0.62 (0.41, 0.93)	0.023
Provider-related variables		
Referring Source		
** **Outpatient	Reference	
** **Inpatient	1.57 (0.12, 21.46)	0.734
** **PCHP	3.05 (0.22, 42.23)	0.405
Referring provider gender		
** **Male	Reference	
** **Female	1.43 (0.97, 2.11)	0.071
** **Team	0.34 (0.03, 4.71)	0.425
Referring provider title		
** **Attending physician	Reference	
** **NP/PA	0.63 (0.36, 1.09)	0.095
** **Trainee (resident or fellow)	0.84 (0.49, 1.43)	0.523

System-level factors, including referral source and referring provider characteristics, were also assessed for their association with hospice LOS. Though patients referred from the outpatient setting had a longer median LOS than those referred from the hospital, there was no statistically significant difference in the odds of short or long LOS according to referral source. Referring provider type and gender, however, did have statistically significant associations with hospice LOS. Compared with attending physicians, patients referred by advanced practice providers had greater odds of short hospice LOS (OR: 1.73; 95% CI: 1.04–2.88) Patients referred to hospice by a female clinician had lower odds of short hospice LOS (OR: 0.63, 95% CI: 0.42, 0.96) compared with those referred by male clinicians. There was no difference in the odds of short or long hospice LOS among patients referred by trainee physicians or inpatient clinical teams than patients referred by outpatient attending physicians.

## Discussion

Identification of factors associated with short hospice LOS is an important step toward overcoming barriers to hospice utilization and timely hospice referrals. Within our study, the factor most strongly associated with hospice LOS was the primary diagnosis for hospice referral. Consistent with prior studies of hospice care, patients included in our cohort had a predominance of cancer as their primary hospice diagnosis.^15,16^ Compared with patients referred to hospice for cancer, patients referred for stroke, heart disease, and other causes had 6.45-, 1.4-, and 1.6-fold greater odds, respectively, of being enrolled in hospice for less than 7 days. Importantly, patients with dementia, heart disease, lung disease, and other hospice referral diagnoses also had greater odds of being enrolled in hospice for longer than 90 days, with odds ratios of 2.3, 2.5, 3.2, and 2.1, respectively. These findings reflect the bimodal distribution of prognosis of chronic diseases such as heart failure and chronic obstructive pulmonary disease, the less predictable course of these conditions as compared with cancer, and the lack of integration of palliative care services into cardiovascular and pulmonary specialties as into oncology practices. While there has been a lot of attention paid to early integration of palliative care into oncology practices, no such efforts were made for other health conditions.^8,17^ As a result, patients with advanced noncancer diagnoses can have greater difficulty accessing palliative care, as their clinicians struggle to predict their disease course and life expectancy and have less experience with hospice care and referrals overall.^18^ Ultimately, the integration of palliative care into other specialties, in the way that the ASCO has advocated for patients with cancer, should be considered for other disciplines to improve the timeliness of hospice referrals and allow patients and caregivers to derive meaningful benefit from this service. Specialists and primary care clinicians would also benefit from focused education about palliative care and hospice during residency and fellowship training, and health systems should consider integrating palliative care services into all service lines and not just oncology.

We also found that patients living in long-term care facilities prior to hospice enrollment had greater odds of short hospice LOS and lower odds of a long hospice LOS than patients living in the community. There are several potential explanations for this, each calling for greater awareness of hospice services and potential for goal-concordant, symptom-driven care for patients at the end of life. First, clinicians and caregivers may be less likely to seek hospice care for patients they perceive as already receiving supportive services in a long-term care facility. However, hospice provides patients with care that far exceeds what is usually available in skilled nursing facilities, including close monitoring for and treatment of pain and discomfort, psychological and spiritual support for patients and caregivers, and more. Second, patients who live in long-term care facilities may have fewer contacts with physicians, particularly specialists, limiting the opportunities for goals of care conversations, advance care planning, and possible hospice referral. Finally, long-term care residents are likely frailer and more ill than patients living in ambulatory settings and, as a result, pass away quicker in hospice, particularly if their referral to hospice is delayed. These results call for greater presence of palliative medicine and hospice services in long-term care facilities, so advance care planning can be optimized in this setting. To this end, contractual agreements and even common ownership between long-term care facilities and hospice agencies have grown in recent years. Long-term care patients receiving hospice care from co-owned agencies have even experienced an increased likelihood of a long hospice LOS.^19^ Further research is needed to determine if this practice durably improves hospice referral timing as well as patient and family-reported outcomes among long-term care facility residents. An additional strategy could involve increasing the presence of palliative medicine services at long-term care facilities through telemedicine and/or a consult team.

There were also significant associations between clinician type and gender and hospice LOS. Being referred to hospice by a nurse practitioner or physician assistant was associated with greater odds of short hospice LOS. It is possible that advanced practice providers may not feel as comfortable initiating hospice referrals, or they may defer goals of care decision making to attending physicians. While advanced practice providers in Mayo Clinic primary care practices maintain their own independent patient panels and, as such, are the primary health care providers for them, they may take a more co-management role with physicians in specialty practices. With the growing number of advanced practice providers functioning independently in the primary care setting, hospitals, and different medical specialties, they must receive appropriate training for caring for patients at their end of life. Ensuring that clinicians across the health system continuum are familiar with hospice eligibility, have goals of care and advance care planning discussions with patients and families, and are comfortable initiating referrals is essential for improving the timing of hospice enrollment and optimization of LOS.

We also found that patients referred by female clinicians had 37% lower odds of being enrolled in hospice for less than 7 days than those referred by male clinicians, with no difference between female and male referring clinicians with respect to having a very long LOS. This finding may reflect differences in communication styles, as female primary care providers have been shown to use more patient-centered language, with more frequent emotionally focused discussion, positive reinforcement, and active listening.^20^ As such, female clinicians may be more likely to engage patients in shared decision-making about their goals of care and support advance care planning early in the course of the patient’s end-of-life care, ultimately making more timely referrals to hospice. These data are also consistent with other work demonstrating lower 30-day mortality and 30-day readmission rates for patients treated by female internists in the hospital.^21^ While our linear model is unable to tease out complex interdependencies between variables, such as provider gender and title or practice setting, and therefore cannot define the phenotype of the optimal clinician, to our knowledge this is the first study identifying provider gender differences impacting hospice enrollment.^22^

While our study was not designed to evaluate the direct impact of the Palliative Care Homebound Program on hospice referrals or LOS, we found that patients in this program had a much longer median hospice LOS (43 days) than patients referred from other ambulatory settings (28 days) or the hospital setting (9 days). The PCHP is a multidisciplinary program designed for older adults with complex health needs and focuses on palliative care and symptom management, though often in addition to curative treatment and/or not eligible for hospice. This program was previously shown to be cost-effective and associated with fewer hospitalizations and higher rates of advance care planning completion, which likely explains the higher odds of timely hospice referrals and longer hospice LOS that we saw in our study.^12,23^ Other programs that deliver home-based palliative care have also been demonstrated to result in higher hospice utilization with trends towards longer hospice stays.^12,23^ While at the time of the PCHP’s inception, there were concerns that it would delay or hinder hospice referrals and adversely affect hospice LOS, our data confirm this is not the case.

An important limitation of this study is the lack of racial, ethnic, and geographic diversity of our patient population. Within our study population, 95% of patients were non-Hispanic White, compared to 82.5% of Olmsted County residents.^24^ While we were not able to detect differences in hospice LOS between White patients and patients from racial and ethnic minoritized backgrounds, the underrepresentation of non-White patients in the cohort itself raises concerns about inadequate rates of hospice referrals overall. This reflects recent research showing racial disparities in end-of-life care, with Black patients less likely to use hospice and more likely to visit the emergency department and receive intensive care.^13,14^ These disparities have also been linked to increased health care spending for non-White patients at the end of life.^25^ Further research is needed to investigate patterns of hospice referral among diverse populations to better understand barriers to quality end-of-life care.

Other limitations include the fact that hospice referrals were generated from a single integrated health care delivery system and that we solely included nonprofit hospice organizations. This could have impacted the distribution of primary hospice diagnoses and LOS, as Medicare’s per diem payment structure may underlie the demonstrated preference for patients with low need and longer LOS within the for-profit hospice model.^26^ We also did not collect data on insurance status due to institutional limitations. Insurance status could influence both access to hospice care and LOS. Additionally, our findings may be influenced by grouping hospice diagnoses in categories such as “heart disease” or “cancer” rather than evaluating each diagnosis individually. Such groups are heterogeneous and may have very different life expectancies and hospice LOS, such as in the example of cancer, where patients with hematological cancers may experience shorter LOS than those with solid tumors. This approach may have obscured potential correlations between our variables and thus impacted regression model assumptions.

Nevertheless, this is the first study to rigorously examine patient- and system-level factors associated with hospice LOS using data from both the clinical setting and independent hospice agencies. As such, it provides valuable information on hospice referral practices, highlights the role of hospice care in both inpatient and outpatient care delivery, and calls for greater focus on timely and equitable referrals to hospice.

## Conclusions

In this single-center study examining hospice LOS among adult patients referred to nonprofit hospice agencies in the United States, we found marked variation in the duration of hospice enrollment as a function of the primary referral diagnosis. While the median duration of hospice enrollment was 18 days, 31.4% of patients were enrolled up to 7 days and 15.3% were enrolled longer than 90 days. Patients with stroke had the shortest median LOS, while patients with heart disease had the longest. Overall, patients who lived in long-term care facilities (vs. community-dwelling), those referred by an advanced practice provider (vs. attending physician), and those referred by male clinicians (vs. female clinicians) had higher odds of being enrolled in hospice less than 7 days. Increased awareness about hospice eligibility and referral requirements, support for advance care planning, and integration of palliative care into primary care and subspecialty practices may be effective strategies to improve hospice LOS.

## Abbreviations Used

ALF: Assisted Living Facility
ASCO: American Society of Clinical Oncology
CIMGP: Community Internal Medicine, Geriatrics, and Palliative Care
CMS: Nurse Practitioner
LOS: length of stay
LTC: Long Term Care
NHPCO: National Hospice and Palliative Care Organization
NP: Nurse Practitioner
PA: Physician Assistant
PCHP: Palliative Care Homebound Program
